# Naturalistic speeding data: Drivers aged 75 years and older

**DOI:** 10.1016/j.dib.2016.05.016

**Published:** 2016-05-16

**Authors:** Anna Chevalier, Aran John Chevalier, Elizabeth Clarke, John Wall, Kristy Coxon, Julie Brown, Rebecca Ivers, Lisa Keay

**Affiliations:** a The George Institute for Global Health, Sydney Medical School, The University of Sydney, GPO Box 5389, Sydney, NSW 2001, Australia; b Safer Roads Consulting, 53 Lachlan St, Thirroul, NSW 2515, Australia; c Kolling Institute of Medical Research, Sydney Medical School, The University of Sydney, Level 10, Kolling Building 6, Royal North Shore Hospital, St Leonards, NSW 2065, Australia; d The Centre for Road Safety, Transport for NSW, Level 3, 84 Crown St, Wollongong, NSW 2500, Australia; e School of Science and Health, Western Sydney University, Narellan Road Campbelltown, NSW 2560, Australia; f Neuroscience Research Australia (NeuRA), Margarete Ainsworth Building, Barker St, Randwick, NSW 2031, Australia

**Keywords:** Older drivers, Speed, Road safety, Naturalistic, In-vehicle monitoring, Device

## Abstract

The data presented in this article are related to the research article entitled “A longitudinal investigation of the predictors of older drivers׳ speeding behavior” (Chevalier et al., 2016) [1], wherein these speed events were used to investigate older drivers speeding behavior and the influence of cognition, vision, functional decline, and self-reported citations and crashes on speeding behavior over a year of driving. Naturalistic speeding behavior data were collected for up to 52 weeks from volunteer drivers aged 75–94 years (median 80 years, 52% male) living in the suburban outskirts of Sydney. Driving data were collected using an in-vehicle monitoring device. Global Positioning System (GPS) data were recorded at each second and determined driving speed through triangulation of satellite collected location data. Driving speed data were linked with mapped speed zone data based on a service-provider database. To measure speeding behavior, speed events were defined as driving 1 km/h or more, with a 3% tolerance, above a single speed limit, averaged over 30 s. The data contains a row per 124,374 speed events. This article contains information about data processing and quality control.

**Specifications Table**

**Table:** 

Subject area	Road safety
More specific subject area	Speeding; older drivers
Type of data	Tables, figure
How data were acquired	The in-vehicle monitoring device consisted of a C4D Data Recorder with an external GPS receiver. The hardware included an internal tachograph, real-time clock, 128 MB of flash memory and internal battery (1300 mA). The GPS data were recorded at 1 Hz (each second) and determined driving speed through triangulation of satellite collected data. These data were linked with supplier-provided mapped speed zone data
Data format	Processed, assessed for quality control
Experimental factors	GPS data were linked with speed zone data
Experimental features	The definition developed for speed events and steps taken to process data to identify and validate these events are detailed below
Data source location	North-West Sydney
Data accessibility	The dataset is within this article

**Value of the data**

• Naturalistic methods are being used increasingly in road safety research, but little is known about the distribution of this type of data. The data provided in this manuscript may be used to calculate sample sizes for other studies investigating speeding behavior.
• Methodological considerations are reported including monitoring inactivity and quality control.
• This data could be considered for use in future meta-analysis combining this data about older drivers׳ speeding behavior with other datasets which include a broader range of age groups and other settings.

## 1 Data

**Fig. 1:**
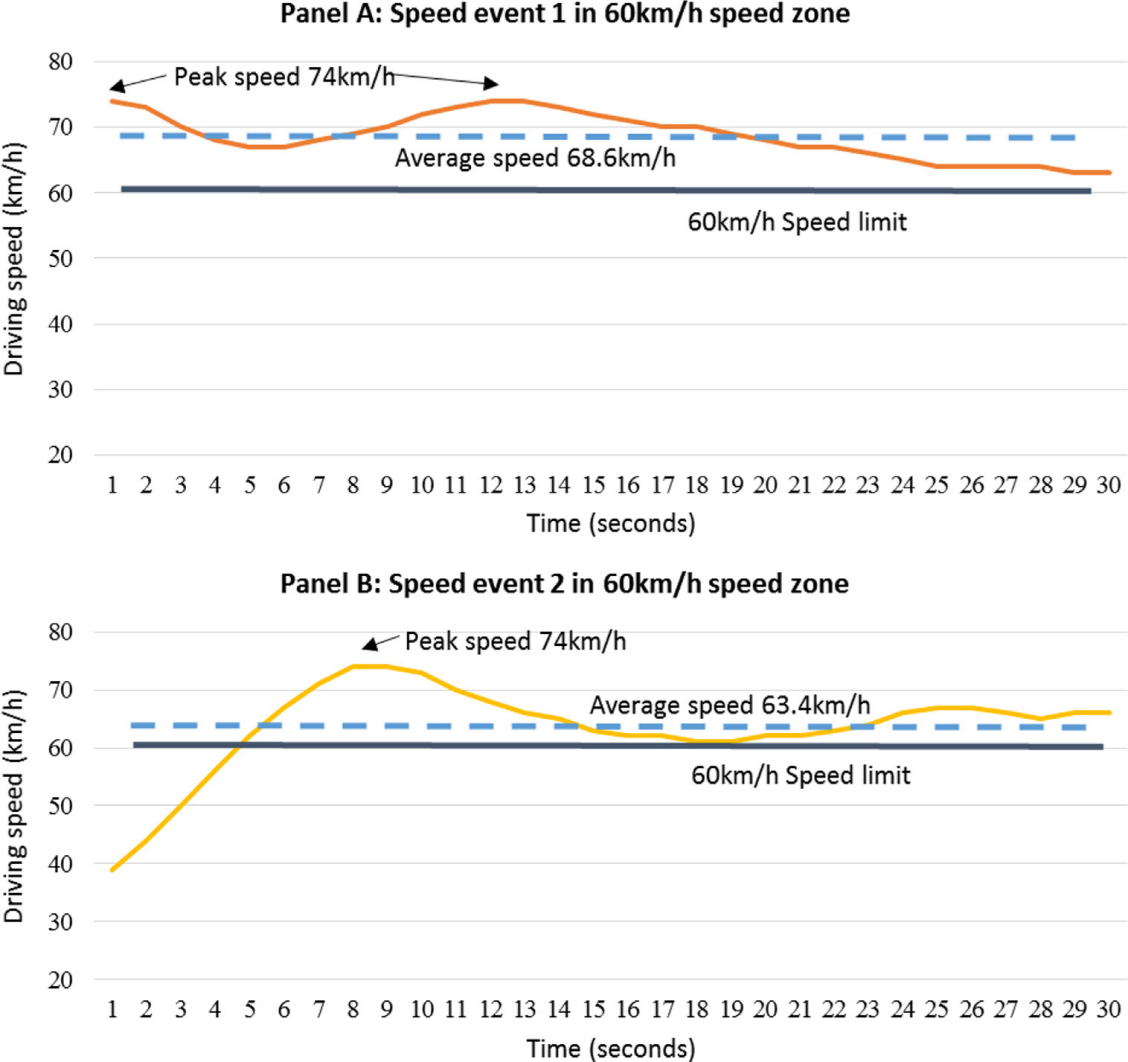
Two 30 s duration speed events that occurred in 60 km/h speed zones, panel A shows all speed recordings being above 60 km/h and panel B shows some speed recordings being below 60 km/h with the average being greater than the speed limit in accordance with the speed event definition.

The dataset contains a row per speed event (Supplementary Table 1). The variables within the dataset are described in Table 1. Fig. 1 depicts variability within two speed events that occurred in 60 km/h speed zones.

## 2 Experimental design, materials and methods

### 2.1 Participants

Volunteer participants were from the control group of a randomized control trial (*n*=380) [2] who agreed to have their vehicle instrumented (*n*=182/190). Participants were aged 75–94 years (median 80 years) and 52% (95/182) were male. Participants resided in the urban outskirts of north-west Sydney (in the Hills, Hornsby, Kur-ring-gai and Parramatta Local Government Areas); held a driver׳s license; owned and were the primary driver of a vehicle (undertaking greater than 80% of driving). Participants were excluded if they received greater than two errors on the Short Portable Mental Status Questionnaire cognitive assessment [3]. Data were collected between July 2012 and May 2014. The in-vehicle monitoring data has been linked to demographic information about participants in other analyses [1], [4].

### 2.2 Data acquisition

The C4D Data Recorder was approximately 11 cm x 8.5 cm x 3 cm in size with an external GPS antenna. The hardware was integrated into the vehicle electrical system using a custom-designed cable loom. This cable integrated the unit and vehicle, providing a fused connection to the ignition and power source, as well as electrically grounding the vehicle. The device powered on and off with the vehicle ignition. The device transmitted captured data via the mobile telecommunications network every 20 s while the vehicle was running. Data were transmitted to a secure service-provider server. Prior to uploading data each week to the researcher׳s secure server, the provider linked the GPS data with mapped speed zone data sourced from the provider׳s established, maintained database of speed limits across the national road network. To protect data confidentiality, both servers restricted staff access.

Weekly datasets included a non-aggregated file of GPS data recorded at 1 Hz, and a mapped kmz format file for each participant trip. The kmz files opened in Google Earth (Google Earth *software (version 7.1.5.1557*) 2015, Google Inc.: Mountain View, CA, US) and depicted the non-aggregated GPS data for each participant trip, including start and end locations, route driven, direction of travel (arrows) and exceeding the speed limit (shown in red, or green for driving above or at/below the speed zone respectively). These data were used to manually examine validity of individual trip data where necessary.

### 2.3 Device malfunctions

To identify device malfunctions, inactivity was monitored approximately every three days. Inactive devices were sent a Short Message Service (SMS) by the service-provider and asked to respond. If the device was inactive for 14 days the participant was contacted by the study team. This strategy was employed to reduce the number of phone calls to infrequent drivers. During a typical month, approximately 2% (2/114) of installed control group devices were identified as inactive and 8 inactivity tests were conducted. In the majority of cases, the participant reported not driving (due to vacation, illness, rain, injury or vehicle breakdown). If a device was identified as not recording driving, a technician made a maintenance visit to fix or replace the device, which only occurred approximately 8 times for the control group during our study. In instances where a participant may have been driving or staying in a remote area without General Packet Radio Service (GPRS) coverage (over which the data were transmitted), the data would be stored on the device and uploaded to the server upon their return to an area with coverage.

### 2.4 Speed event definition

We developed criteria to define speed events for use as a measure of speeding behavior, based on the concept of point-to-point speeding. Speed events were identified from a comparison of average driving speed with a service-provider database of mapped speed limits (Speed Alert by Smart Car Technologies Pty Ltd). To minimize identification of false positive events, speed events were defined as driving at an average 1 km/h or more above the speed limit plus 3% tolerance, and were 30 s in duration. The 3% tolerance was applied to account for inaccuracies in the measuring equipment or participant׳s speedometer and therefore, the driver׳s perception of speeding. The 30-s duration was applied as average speed remains elevated too long to define the end of an event based on traveling at or below the speed limit. An event was required to occur in a single speed zone (minimum 40 km/h). Events drew upon a minimum three satellites per record to triangulate speed. Events contained a minimum of 25 GPS records in 30 s, to permit missing GPS records. If no event was identified, the search re-commenced at the next record.

Within this definition, (i) not all speeding has been captured, as the behavior must meet the criteria; (ii) vehicle speeds may at times drop below the speed limit, although the average over the 30 s duration will be above the speed limit (Fig. 1), (iii) speeding undertaken during GPS drop out would not be captured unless it meets the criteria which allows for some GPS drop out, as is common with GPS data.

An audit of a random sample of 300 speed events investigated agreement between the Speed Alert database and the speed zone estimated by Google Maps API. The audit revealed high levels of agreement (87%, 262/300). However, there was systematic underestimation of the speed zone for high range speed events, related to the Speed Alert database mistakenly allocating a local road speed limit to segments of travel on high speed roads such as motorways. A second source of inaccuracy was in identifying the 40 km/h speed limits, which only operate before and after school times. Therefore, speed events were limited to those involving speeding <10 km/h above the speed limit plus the tolerance and occurring in speed zones of 50–110 km/h. The rate of agreement in this dataset was 92% (245/266).

### 2.5 Processing data

Due to the large volume of data, robust programs were required to maintain the integrity, and confirm the validity of the data by (i) ensuring each file was attributed to the correct device, participant and date; (ii) thoroughly checking data as processed to identify errors such as missing files and missing rows of data; and (iii) automating processing to reduce the need for manual handling of data, processing time and incidence of human error.

Data were provided for all participants by project week. Matlab (MathsWorks, MatLab software R2012a, Natick, MA, US) programs were developed to copy these data into program-created folders for individual participants by their week of involvement in the study (participant week). To identify the relevant participant weeks, the program referred to a mapping csv file containing unique participant and device identifiers, as well as device installation dates. The programs identified when a participant had more than one device during a single week, identified the order of device installation, and re-numbered trips for the week sequentially.

### 2.6 Data quality control

Where a participant reported they had lent their vehicle to a relative or friend, data for this period were replaced with missing values. Data from malfunctioning devices were also replaced with missing values.

Validity tests of speed events included assessing the definition parameters were met. These included: the event involved driving an average of 1 km/h plus a 3% tolerance above the speed limit over 30 s; the event occurred in a single, viable speed zone (minimum 50 km/h and maximum 110 km/h); the event contained a minimum 25 GPS records in 30 s; and each record within the event drew upon three or more satellites. In addition, to ensure data were captured accurately, speed events/100 km and peak travel speeds were assessed as to their reasonableness. We reviewed mapped trip or raw data files for any unusual data to determine validity. After undertaking these data quality control tests, 98% (124374/127047) of speed events were considered valid.

## Figures and Tables

**Table 1:** Description of variables in valid speed event dataset.

Variable name	Description
Partid	Participant identification number
Partweekno	Participant week number
Speedeventno_valid	Speed event number
Averagespeed	Average speed during event (km/h)
Speedzone	Speed limit (merged from third party database of speed limits on road network)
Date	Date (yyyymmdd) (derived from Unix timestamp)
Time	Time (hh:mm:ss, 24 h) (derived from Unix timestamp)
Minsatforduration	Minimum number of satellites for duration of event (quality control measure)
Gpsrecords	Number of GPS records (quality control measure)
